# Epigenetic regulation of HIV-1 latency: focus on polycomb group (PcG) proteins

**DOI:** 10.1186/s13148-018-0441-z

**Published:** 2018-02-05

**Authors:** Sheraz Khan, Mazhar Iqbal, Muhammad Tariq, Shahid M. Baig, Wasim Abbas

**Affiliations:** 1 Health Biotechnology Division (HBD), National Institute for Biotechnology and Genetic Engineering (NIBGE), PO Box 577, Jhang road, Faisalabad, 38000 Pakistan; 2 Pakistan Institute of Engineering and Applied Sciences (PIEAS), Nilore, Islamabad, Pakistan; 3 Department of Biology (Epigenetics group), SBA School of Science and Engineering, LUMS, Lahore, 54792 Pakistan

**Keywords:** HIV-1 latency, Polycomb group (PcG) proteins, Epigenetics, HIV-1 reservoirs, Histone modification

## Abstract

HIV-1 latency allows the virus to persist until reactivation, in a transcriptionally silent form in its cellular reservoirs despite the presence of effective cART. Such viral persistence represents a major barrier to HIV eradication since treatment interruption leads to rebound plasma viremia. Polycomb group (PcG) proteins have recently got a considerable attention in regulating HIV-1 post-integration latency as they are involved in the repression of proviral gene expression through the methylation of histones. This epigenetic regulation plays an important role in the establishment and maintenance of HIV-1 latency. In fact, PcG proteins act in complexes and modulate the epigenetic signatures of integrated HIV-1 promoter. Key role played by PcG proteins in the molecular control of HIV-1 latency has led to hypothesize that PcG proteins may represent a valuable target for future HIV-1 therapy in purging HIV-1 reservoirs. In this regard, various small molecules have been synthesized or explored to specifically block the epigenetic activity of PcG. In this review, we will highlight the possible therapeutic approaches to achieve either a functional or sterilizing cure of HIV-1 infection with special focus on histone methylation by PcG proteins together with current and novel pharmacological approaches to reactivate HIV-1 from latency that could ultimately lead towards a better clearance of viral latent reservoirs.

## Background

Over the last two decades, combination antiretroviral therapy (cART) has dramatically improved the management of human immunodeficiency virus type 1 (HIV-1) infection and remarkably declined the morbidity and mortality associated with human immunodeficiency virus (HIV)/acquired immunodeficiency syndrome (AIDS) [1]. Anti-HIV drugs particularly cART suppress plasma viremia below the detection limit (< 50 copies/ml). Six classes of antiretroviral drugs currently exist, and each class targets a different step in the viral life cycle [2]. Despite the administration of cART, it is still impossible to eliminate HIV in infected individuals. Highly sensitive methods always detect residual viremia in HIV-1-infected subjects on cART. Moreover, rebound viremia occurs when cART is interrupted [3, 4]. It is generally believed that the rebound viremia occurs from latent viral reservoirs such as resting CD4+ T cells (more specifically central memory CD4+ T cells) and cells from monocyte-macrophage lineage including microglial cells, i.e., the resident macrophages of central nervous system (CNS) [5, 6]. It seems that sterilizing or functional cure of HIV-1 is not possible with current antiretroviral regimen. Various limitations are associated with current cART [7, 8]. Lifelong adherence to cART is required to suppress the viremia, while rebound viremia occurred after cART interruption [4, 9]. Moreover, there are severe side effects of cART treatment such as neurocognitive and metabolic disorders [8, 10]. Most of the time, HIV-drug resistant strains emerge due to high mutability of the virus that limit and complicate the treatment options [11].

Rebound viremia from the Mississippi baby and Boston patients suggest that functional cure for HIV will be difficult to achieve. It may be due to the presence of latent viral reservoirs [12, 13]. Latently infected resting CD4+ T cells and macrophages are major viral reservoirs in HIV-1 infection. The latent viral reservoirs persist in these long-lived cells that harbor integrated HIV-1 DNA, but remain transcriptionally silent, and are therefore hidden from immune surveillance. Hence, they cannot be targeted by cART [14–16]. In addition, IL-7 drives homeostatic proliferation of latently infected resting memory CD4+ T cells and maintains this pool of cells for many years [17]. Further, the latent viral reservoirs are established during early infections and pose a significant barrier to HIV-1 eradication strategies [18–20]. The early treatment with cART can reduce the size of these latently infected viral reservoirs, but it cannot prevent the establishment and maintenance of HIV-1 persistence as seen in the Mississippi baby [21].

HIV-1 transcription is often silenced by epigenetic changes in the residual reservoirs under cART [22, 23]. These epigenetic changes remain a principle obstacle in eradication and cure of HIV/AIDS [24]. Understanding the molecular mechanisms involved in the establishment and maintenance of HIV-1 latency is of prime importance in order to clear or achieve small population of latent reservoirs [25]. Significant progress has been made in the development of various anti-HIV therapies that may target HIV and prevent the disease progression. It includes therapeutic vaccine (viral vectors and DNA-based vaccines), cell-based therapies (adoptive T cell therapy and chimeric antigen receptors), gene therapies (genetically modified stem cells), broadly neutralizing antibodies (bnAbs), and epi-drugs (viral latency reversing or promoting agents) [26–31]. However, latency-reversing or latency-promoting agents that eliminate or suppress the latently infected cells have received much attention [32, 33]. Current report indicates that histone methylation by Polycomb group (PcG) proteins affect HIV latency in early phases of infection [34]. Originally, PcG proteins are known as transcriptional repressors that epigenetically alter chromatin and are involved in the maintenance and establishment of cell fate [35, 36]. In this review, we will discuss the main mechanisms involved in the establishment and maintenance of HIV-1 latency, with a focus on PcG proteins. Latency-reversing or latency-promoting approaches together with effective therapeutic agents that constantly enhance immune response to HIV-1 infection may be helpful to achieve a sterilizing or functional cure.

## HIV-1 latency

### Pre- and post-integration latency

**Fig. 1:**
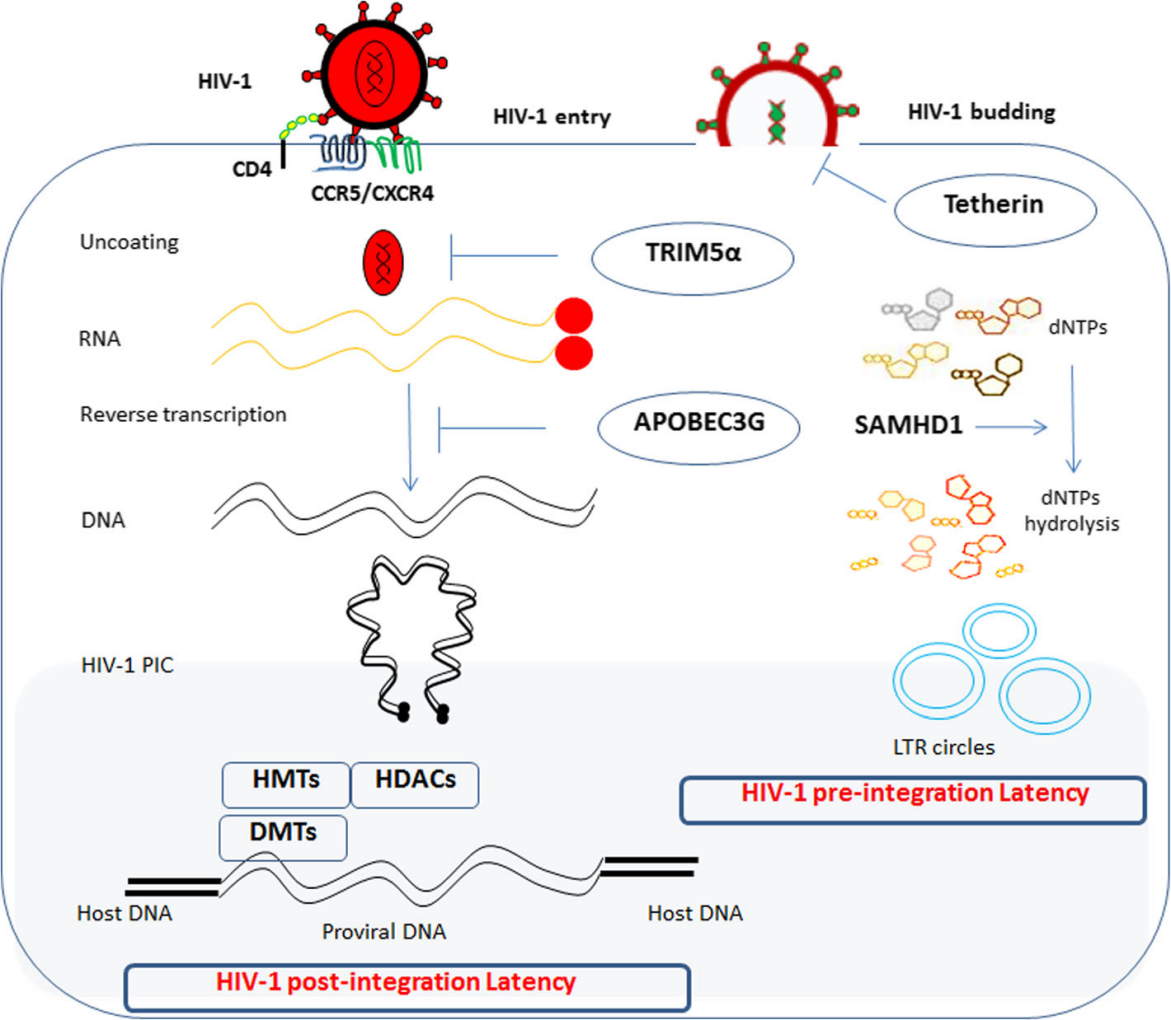
Cellular and virological events in early HIV-1 infection. HIV-1 infects the cells having CD4 receptor and either of the coreceptor CXCR4 and/or CCR5. The resting cells are less permissive to viral infection due to low metabolism characterized by low level of available dNTPs for energy source and reverse transcription. Various cellular intrinsic factors influence HIV-1 infection process. TRIM5α targets viral capsid and interferes with uncoating process of viral core. Cytidine deaminases, APOBEC3G, or F cause mutation in viral DNA resulting virus inactivation. Tetherin arrests viral particles on the cell membrane and inhibits virion budding. SAMHD1 inhibits viral infection by depleting intracellular pool of dNTPs. Pre-integration latency refers to unintegrated form of HIV-1 genome. In post-integration latency, the integrated form of proviral DNA is silenced by various DNA and chromatin-modifying enzymes

HIV-1 latency can be divided into pre-integration and post-integration latency depending upon whether or not the virus has been integrated into host genome [20, 23, 37]. Pre-integration latency occurs from partial or complete inhibition of viral life cycle (prior to integration of viral DNA into host genome) at one or several of the stages: incomplete reverse transcription process of viral RNA, low metabolic state of host cell, presence of host restriction factors such as tripartite motif-containing protein 5α (TRIM5α) and apolipoprotein B mRNA-editing enzyme catalytic subunit 3G (APOBEC3G), and blockage of import of pre-integration complex (PIC) into nucleus (Fig. 1) [23]. In CD4+ T cells, pre-integration latency does not account for establishment of HIV-1 latency for long duration. In contrast to CD4+ T cells, tissue macrophages may harbor an unintegrated form of HIV for a longer period [37, 38]. However, pre-integration latency is clinically less important and less relevant in HIV-1 eradication strategies.

Therefore, the focus of this review is the post-integration latency that occurs when a provirus fails to transcribe its genome and is reversibly silenced after integration into host cell DNA. Such mechanism of viral latency has been documented in HIV-1-infected patients predominantly in resting memory CD4+ T cells, and cells of myeloid lineage such as monocytes/macrophages [39]. However, silencing mechanisms of integrated provirus have been poorly understood; therefore, it is an active area of current HIV-1 research. The epigenetic silencing of provirus depends upon several factors: site and orientation of integration, availability of cellular or host transcription factors, chromatin organization of promoter, viral protein trans-activator of transcription (Tat) and its host-associated factors, and miRNAs. HIV-1 latency operates at several transcriptional and post-transcriptional levels [23]. HIV-1 integrates into host chromosomes in a non-random fashion, mainly intronic regions of actively transcribed genes [40]. A variety of stimuli or inducers such as antigens, mitogens/phorbol esters, and cytokines can activate HIV-1 from post-integration latency [41].

### Chromatin organization and epigenetic players in HIV-1 latency

**Fig. 2:**
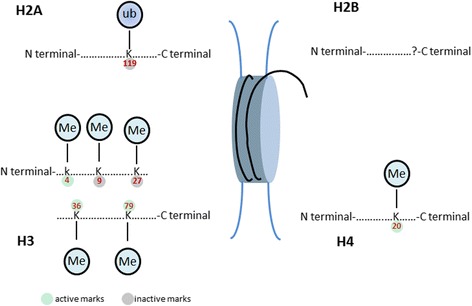
Histone methylation pattern in HIV-1 transcription and latency: histone can undergo post-translational (methylation) modifications. These modifications could determine the gene expression by regulating the local and global chromatin architecture. Trimethylation marks of lysine 4, 36, or 79 on H3 results in gene activation while di or trimethylation of lysine 9 and trimethylation of lysine 27 on H3 is associated with chromatin condensation

The chromatin architecture is critical for the control of eukaryotic gene expression since it modulates the accessibility of cellular transcriptional factors that bind to DNA [42, 43]. Eukaryotic DNA is packaged within the chromatin; and a nucleosome is a functional and structural unit of chromatin. A nucleosome consists of two molecules; each contains four core histones, H2A, H2B, H3, and H4 (Fig. 2) [44]. The amino terminal domain of each histone portrays outside of the nucleosome core and is subject to various types of post-translational modifications. Each nucleosome is wrapped in 146 bp of DNA which is tightly packed in 1.65 super helical turn around the octamer [43, 45]. The nucleosomes are linked to each other by a small fragment of linker DNA which is stabilized by H1 histone. Further, decondensed genome or euchromatin is reported to be linked to actively transcribed genes, while the condensed or heterochromatin to transcriptionally inactive region of genome [43]. The chromatin condensation or decondensation status can be altered through a variety of post-translational modifications [23, 43].

Histone modifications are all reversible and include methylation, acetylation, sumoylation, ADP ribosylation ubiquitination, and phosphorylation [46]. However, we will focus on histone methylation that is one of the most important histone marks for HIV-1 repression mediated by PcG proteins. Histone lysine methyltransferases (HKMTs) catalyze the transfer of methyl groups from the *S*-adenosylmethionine (SAM) to lysine residue. Histone methylation has no effect on histone-DNA interactions rather it provides the platform for the recruitment of various chromatin-modifying enzyme complexes [46–48]. In contrast to acetylation, histone methylation alters the steric and hydrophobic properties of histone which is usually associated with activation as well as repression of transcription. The levels of histone methylation can be mono-, di-, or tri-methylated, depending upon particular functional properties of the associated methyltransferase. The major histone methylation marks include lysine 4, 9, 27, 36, and 79 of H3 and lysine 20 of H4. Generally, H3K4, H3K36, and H3K79 methylation are linked to euchromatin (transcriptionally active) while H3K9, H3K27, and H4K20 methylation are linked to heterochromatin (transcriptionally inactive) (Fig. 2) [49–51]. Moreover, H3K9 di- and trimethylation are associated with transcription repression while H3K9 monomethylation is a transcriptional activation mark. Histone methyltransferases are histone-modifying enzymes which catalyze the transfer of methyl groups to lysine or arginine residues of histone proteins. It is a reversible process, and histone demethylases actively remove the methyl groups [45]. The discovery of histone lysine methyltransferase (HMT), suppressor of variegation 3-9 homolog 1 (Suv39h1), has increased our understanding of gene expression regulated by histone methylation [52, 53]. The Suv39h1 is conserved from yeast to human, and its homolog in Drosophila is Su(var) 3-9 [54, 55]. HMTs have been characterized by the presence of SET (Su(var)3-9, Enhancer of Zeste, Trithorax) domain. The SET domain contains catalytic HMT activity [56]. Currently, approximately seven SET domain families have been described which are suppressor of variegation 3-9 (Suv39), SET1-2, retinoblastoma protein-interacting zinc-finger (RIZ), enhancer of zeste (EZ), suppressor of variegation 4-20 (Suv4-20), and SET and MYND domain-containing proteins (SMYD) [57]. The differential regulation (activation and repression) and transcription of a gene is mediated through methylation of histone at different residues. Actually, different histone modifications allow the cell to respond through various chromatin-associated proteins which recognize specific modifications on histone residues [49]. For instance, heterochromatin protein 1 (HP1) binds to methyl group on H3K9 leading to gene repression [58]. While transcriptional activator WD repeat-containing protein 5 (WDR5) recognizes methylated H3K4 and promotes gene activation [59]. In contrast to HMT, the HDMs remove methyl groups from histones. In this regard, two classes of HDMs have been reported, i.e., Jumonji C (jmjC) domain-containing proteins and lysine-specific demethylase 1 (LSD1). LSD1 can remove mono- or dimethylation marks on histone while jmjC domain containing an enzyme can remove all three methylation marks [60]. Following the HP1 recruitment to methylated histone, DNA methyltransferases (DNMTs) are then recruited at that site and reinforce inhibitory signal by promoting the methylation of nearby sites on DNA [61]. Though DNA methylation and histone methylation are executed by different enzymes, they maintain a close biological relationship in mediating epigenetic silencing which is further termed as double lock [62, 63].

### Histone methylation and HIV-1 latency

**Fig. 3:**
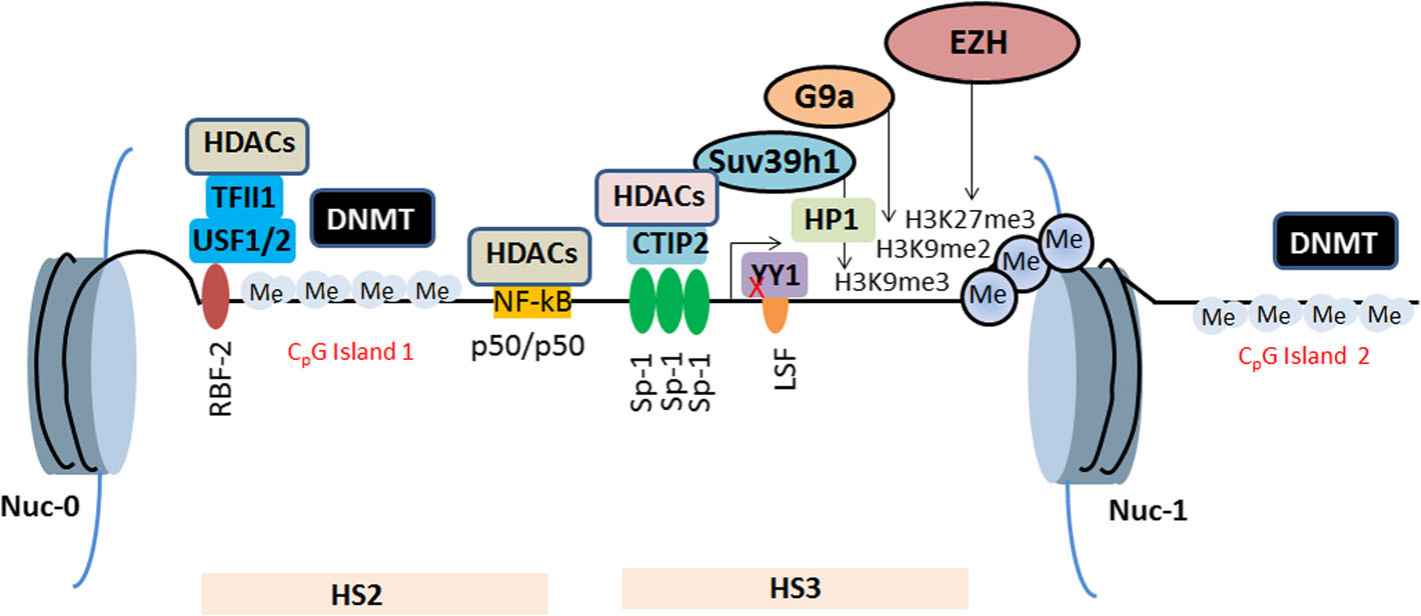
A simplified overview of epigenetic modifications in HIV-1 latency. Several factors regulate HIV-1 latency. HMTs (Suv39h1, G9a, and EZH2) methylate histones at nuc-1. Cellular transcription factors (p50/p50 homodimers, CTIP-2, and YY1 etc) recruit HDACs to 5′ of viral LTR and maintain epigenetically silenced chromatin structure. Sp1 interacts with CTIP-2 and recruits Suv39h1 and HDACs at HIV-1 5′ LTR. Ras-responsive binding factor 2 (RBF 2) is a multicomponent complex that consists of upstream stimulatory factor 1-2 (USF1/2). RBF 2 also contains a multifunctional factor TFII-I. RBF 2 complex recruits HDACs at ras-responsive binding elements (RBEs) and regulates HIV-1 transcription. Two CpG islands flank the HIV-1 transcription start site (TSS) and are methylated by DNMTs in latently infected viral reservoirs

The chromatin organization and epigenetic regulation of HIV-1 promoter are key players in the control of viral transcription and latency. Two nucleosomes (nuc-0 and nuc-1) are precisely positioned on HIV-1 long terminal repeat (LTR) in several latently infected cell lines [42, 64, 65]. The nuc-1 is positioned immediately downstream of transcription start site and imposes a block to transcription initiation and elongation (Fig. 3) [23]. During latency, Tat is absent and only short mRNA-containing trans-activation response elements (TAR) region is transcribed. Nuc-1 is kept epigenetically silenced via several transcriptional factors such as COUP-TF-interacting protein 2 (CTIP-2), Ying-yang 1 (YY1), C-repeat binding factor 1 (CBF-1), and p50/50 homodimers [20, 23, 43, 66, 67]. Following transcriptional activation, nuc-1 is precisely remodeled and is specifically involved in the transcriptional activation process. Transcription factors such as nuclear factor kappa B (NF-kB) (p50/65 heterodimers) and Specificity protein 1 (Sp1) bind to the 5′ HIV-1 LTR and hence enhance the HIV-1 transcription. In addition, viral protein Tat is involved actively in transcriptional process via binding to TAR [23]. Tat interacts with various cellular transcriptional activation factors such as CDK9, cyclin T1, and p300, and thus allows the transcriptional elongation of HIV-1 genes [23, 39].

Suv39h1 and G9a are primarily participated in H3K9me3 and H3K9me2, respectively. These histone modifications have been reported to play an important role in HIV-1 gene silencing in different cellular modeling including primary cells such as peripheral blood mononuclear cells (PBMCs) isolated from HIV-1-infected patients [68]. Actually, Suv39h1 initiates the formation of heterochromatin via recruiting HP1. HP1 protein exists in three isoforms: HP1α, HP1β, and HP1γ. Suv39h1 is required for the deposition of trimethylation marks on H3, and then, trimethylated H3 serves as a platform for HP1, more specifically HP-1γ [69, 70]. Following the knockdown of HP1γ, Suv39h1 is displaced from HIV-1 promoter, and the level of H3K9me3 is also reduced [42, 68]. The HIV-1 LTR is occupied simultaneously by the two transcriptional activators: positive transcription elongation factor b (PTEFb) and P300/CBP-associated factor (PCAF). This results in the activation of viral transcription. Similarly, CTIP-2 also participates in the deposition of H3K9 trimethylation marks [42, 71].

In fact, Sp1 binds to its three binding sites located at 5′ LTR of HIV-1 promoter and recruits histone deacetylases (HDACs) (HDAC1 and HDAC2) to viral promoter via CTIP-2, leading to H3K9 deacetylation. This phenomenon is a prerequisite for Suv39h1-mediated H3K9me3 [71, 72]. Further, Suv39h1 interacts and recruits CTIP-2 at HIV-1 promoter that results in the formation of multi-enzyme complex consisting of HDAC1, HDAC2, and HP1 (HP1α, HP1β, and HP1γ), and thus creates repressive chromatin environment at viral promoter (Fig. 3). Notably, Suv39h1 and CTIP-2 interact functionally to maintain the repressive chromatin environment at HIV-1 LTR. Upon CTIP-2 downregulation, the level of Suv39h1 recruitment and H3K9me3 is decreased with HP1 displacement. [23, 42]. Moreover, Suv39h1-mediated H3K9me3 requires a previous demethylation step of H3K4 by LSD-1. In this regard, CTIP-2 plays a critical role during HIV-1 latency. CTIP-2 interacts with LSD-1, and then this multi-enzyme complex is recruited to HIV-1 LTR. The CTIP-2 and LSD-1 complex then synergistically downregulate the HIV-1 replication and transcription by modulating the epigenetic status of H3K9 in microglial cells, the main target of HIV-1 infection in CNS [73, 74]. In addition to Suv39h1, G9a, another methyl transferase, also regulates HIV-1 latency. Upon the G9a knockdown, the increased LTR or viral transcription and reduced H3K9me2 has been observed. The treatment of BIX01924, a G9a inhibitor, results in decreased H3K9me2 activity, thereby reactivating HIV-1 LTR from latency [42, 75]. Further, G9a-mediated H3K9me2 recruits various enzymatic complexes including HP1 and thereby participating in the development and maintenance of HIV-1 silencing and latency [22]. In addition to H3K9me2 by G9a, Enhancer of Zeste-2 (EZH2) that mediates H3K27me3 is present at high level at the promoter of HIV-1 latent reservoirs [76, 77]. Recently, it has been reported that H3K27me3 of HIV-1 promoter plays an important role in the establishment of HIV-1 latency [77, 78]. During latency, increased H3K27me3 has been observed and thus represents a repressive chromatin structure while EZH2 can be displaced following proviral reactivation in T cells by latency-reversing agents (LRAs) [79]. Several cellular transcription factors such as CBF-1 are responsible for the recruitment of EZH2 at 5′ LTR of HIV-1 promoter. The knockdown of EZH2 results in loss of H3K27me3 and reactivates HIV-1 from latency more strongly than that of Suv39h1 and G9a [42]. EZH2 not only induces HIV-1 latency through H3K27me3 but also serves as a binding plate form of multi-enzyme complex that further suppresses HIV-1 transcription epigenetically. Moreover, EZH2 inhibitor 3-deazaneplanocin A (DZNep) significantly reactivates HIV-1 latent reservoirs while the selective inhibitors of Suv39h1 and G9a have modest effect on HIV-1 latency. These findings have suggested that histone methyltransferase inhibitors (HMTIs) particularly EZH2 inhibitors could represent an attractive and promising therapeutic drug target in the eradication of HIV-1 latent reservoirs [79, 80].

## Polycomb group proteins

**Fig. 4:**
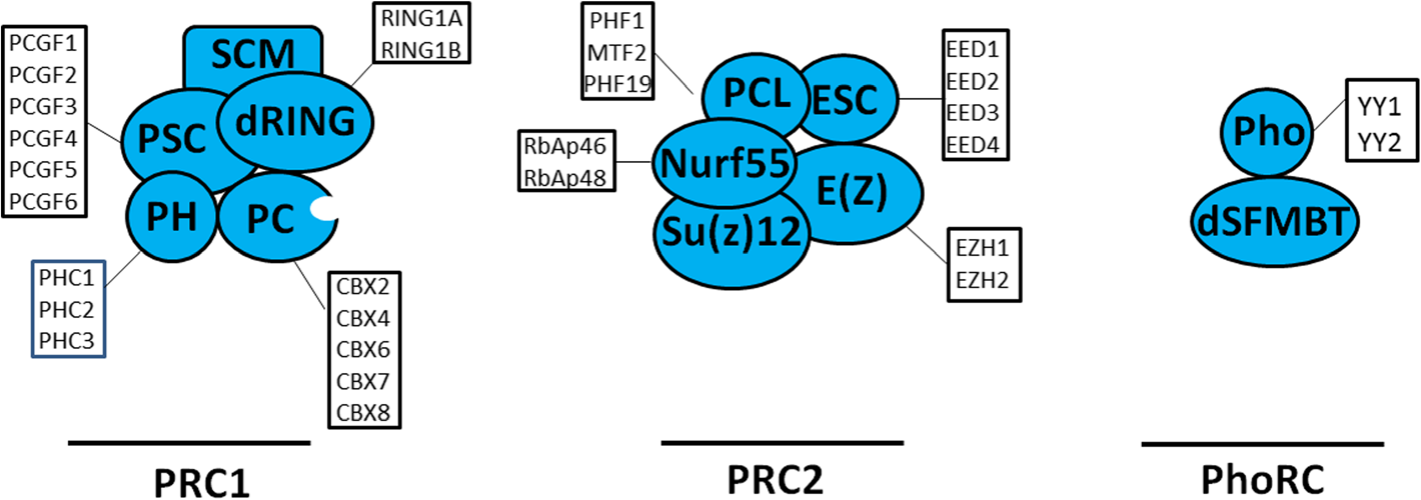
Schematic of types of PcG proteins: the PcG proteins are implicated in transcriptional silencing and formation of higher order chromatin structure. PcG proteins form three main complexes, PRC1, PRC2, and Pho-RC. Three principal PcG complexes have been described in *D. melanogaster*; and the human homologs are also shown. *D. melanogaster* proteins are shown in shapes (blue) while human homologs are drawn adjacent to these. PRC1 and PRC2 have been identified in mammals while PhoRC have only been characterized in *D. melanogaster*

Polycomb genes were initially identified in *Drosophila melanogaster* as the regulators of anterior and posterior body patterns through the repression of Hox genes, which is now they are considered as key regulators and global epigenetic transcriptional repressors of cell fate [81, 82]. Advancement in the recent research have extended our understanding about how the homeotic phenotypes are regulated by polycomb genes [83, 84]. In *D. melanogaster*, members of polycomb group (PcG) proteins exist as multiprotein complex that interacts with chromatin. Also, these complexes mediate the heritable repression of gene expression while the members of Trithorax group (TrxG) activate the same genes [82, 85, 86]. In mammals, three main families of polycomb genes have been identified: Polycomb-repressive complex 1 (PRC1), Polycomb-repressive complex 2 (PRC2), and Pho-repressive complex (PhoRC) (Fig. 4 and Table 1). The PRCs interact with chromatin through Polycomb response elements (PREs). There are hundreds to thousands of PREs which act as binding sites for PRC1 and PRC2 [86–88]. Through different mechanisms, PRCs are recruited at their genomic target sites by CpG islands, non-coding RNAs and transcription factors [89]. The number of polycomb genes identified during evolution from invertebrates to vertebrates rises approximately 20 in *D. melanogaster* to 37 in human and mouse [82, 89]. However, recent data suggests that the variants and diversity of PRCs may be greater than expected [81, 90, 91].

**Table 1:** PcG proteins core complex components in *D. melanogaster* and human

PcG subunits in *Drosophila melanogaster*	PcG subunits in humans	Protein domains	Biochemical functions
Polycomb-repressive complex 1 (PRC1)			
Polyhomeotic proximal (PH-P) and distal (PH-D)	PHC-1/EDR1, PHC-2/EDR2, PHC-3/EDR3	C2-C2 zinc-finger and SAM Domain	Higher order interaction?
Posterior sex comb (PSC)/suppressor 2 of Zeste (Su(z)2)	BMI1/PCGF4 MEL18/PCGF2 NSPC1/PCGF1 RNF110/ZFP144	Ring finger domain (C3HC4 Zinc-finger)	Co-factor for SCE and compacts chromatin
Polycomb (PC)	CBX2/HPC1 CBX4/HPC2 CBX6, CBX7 and CBX8/HPC3	Shadow domain and chromodomain	Binds to H3K27 trimethylation marks and CBX4 is reported to be a SUMO E3-ligase
Sex comb extra (SCE)/dRING	RING1/RING1A, RNF1 RING2/RING1B, RNF2	Ring-finger domain (C3HC4 Zinc-finger)	Ubiquitinates H2AK118 (H2AK119 in vertebrates) and compacts the chromatin
Sex comb on midleg (SCM)	SCMH1 SCML2	MBTs, SAM, DUF3588 and Zinc-finger and SPM domain	?
Polycomb-repressive complex 2 (PRC2)			
Enhancer of Zeste (E(z))	EZH1 and EZH2/KMT6	CXC domain, SET domain, SANT and homolog domain I and II	Catalyzes trimethylation (H3K27)
Suppressor of Zeste 12 (Su(z)12)	SUZ12	C2-H2 Zinc-finger, VEFS Box, alanine rich and Glycine rich	Enhances the enzymatic activities of EZ and also important for nucleosome binding
Extra sex comb (ESC)	EED or WAIT-1	WD-40 repeats	Stimulates H3K27 methyltransferase
Extra sex comb like (ESCL)			
Chromatin assembly factor 1 (Caf1)/nucleosome remodeling factor 55 (Nurf55)	RbAp46/RBBP7 RbAp48/RBBP4	WD-40 repeats	Binds to histones and suppressor of Zeste 12
Polycomb-like (PCL)	PHF1/PCL1 MTF2/PCL2 PHF19/PCL3	PHD finger and tudor domain	Induces trimethylations and recruits PRC2
Pleiohomeotic (Pho)-repressive complex (Pho-RC)			
PHO	YY1, YY2	Zinc-finger	DNA binding
SFMBT (CG16975)		MBT and SAM	Bind to mono- and dimethylated histone at H3K9 and H4K20

### Polycomb-repressive complexes

#### Polycomb-repressive complex 1

**Fig. 5:**
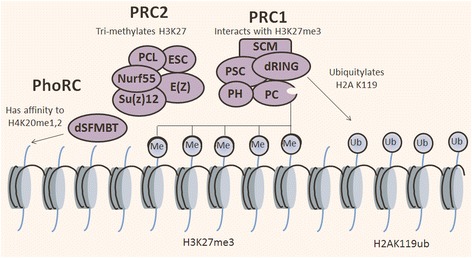
Epigenetic silencing mechanism of PcG proteins. Following the recruitment of PRC2 to chromatin, the histone methyltransferase E(Z) catalyzes trimethylation onto H3K27. Subsequent recruitment of PRC1 occurs through affinity binding of the chromodomain of PC subunit to H3K27me3. The PRC1 dRING monoubiquitylates onto H2K119 which further consolidates the transcriptional repression and enhances the chromatin compaction. Pho-RC comprises of PHO and SFMBT which binds to PRE via DNA binding activity of PHO. PHO then recruits PRC2 which methylate local chromatin

The purified form of PRC1 contains a group of four core proteins: polyhomeotic (PH), posterior sex combs (PSC), polycomb (PC), and sex comb extra (SCE) also termed as dRING (Fig. 4 and Table 1) [92, 93]. Some additional proteins were also purified simultaneously with these components including the elements of multi-protein complexes such as SMRT-related and ecdysone receptor-interacting factor (SMRTER), member of SIN3 family protein (SIN3A), menin-MLL inhibitor-2 (MI-2), and TATA-binding protein-associated factors (TAF)II62, TAFII85, TAFII110, TAFII250, and ZESTE protein [94, 95]. The PRC1 adds ubiquitlytion to histone H2A, and the RING1 protein has monoubiquitylation E3 ligase activity that is specific for the lysine 119 of H2A (H2AK119ub). This activity is also associated with repressive chromatin structure (Fig. 5 and Table 1) [96]. H3K27me3 mark is specifically recognized by the chromo-domain of PC, while chromatin remodeling is inhibited by PH and PSC component of PcG proteins. The primary complexes of PRC1 can be purified simultaneously with sex comb on midleg (SCM) which assigns the recruitment of PRC1 to PREs [97]. The PRC1 proteins particularly SCE and PSC are the constituents of another polycomb complex entitled as dRING-associated factors (dRAF) which contain lysine demethylase-2 (KDM2) [98]. KDM2 belongs to group of JmjC domain-containing histone demethylase and is the single homolog of mammalian KDM2A, KDM2B, and KDM7 [99]. KDM2 has been known to mediate demethylation of H3K4me3 and H3K36me2 while it is also required for efficient ubiquitination of H2A. Moreover, it also acts as an enhancer of PcG protein and suppressor of the Trithorax (Trx) and absent, small, or homeotic discs 1 (Ash1). Actually, Trx and Ash1 belong to TrxG of transcriptional activator proteins, which maintain homeotic gene expression during Drosophila development [82]. The PSC induces the SCE ubiquitin ligase activity; however, it also contains a Jumonji C (JmjC) domain that facilitates the demethylation of H3K36. H3K36 is a signal of gene activation in its methylated state. As a consequence, the PcG proteins integrate the silencing marks while removing the activation marks in chromatin as well, which are introduced by TrxG [100]. Polycomb group genes in Drosophila have closely related homologs that function alternatively during developmental stages, in different tissues or even in the same cell at different target genes. There are two polyhomeotic genes, i.e., polyhomeotic distal (PH-D) and polyhomeotic proximal (PH-P); however, their functions have been poorly investigated. In the same manner, suppressor 2 of zeste (Su(z)2) and PSC are closely related and are said to have partially homolog functions [101, 102]. Two other pair of PcG proteins which have partially or completely overlapping functions are extra sex comb (ESC) and extra sex combs like (ESCL) and polyhomeotic (PHO) and polyhomeotic like (PHOL) [103, 104]. In humans and mice, obviously than *D. melanogaster*, the number of these proteins has other homologs that might function as alternatively in different tissue or at different targets [105].

#### Polycomb-repressive complex 2

The primary core component of polycomb-repressive complex 2 (PRC2) contains four proteins: suppressor of zeste-12 (Su(z)12), extra sex comb (ESC) or alternatively extra sex comb like (ESCL), enhancer of zeste (E(z)), and chromatin assembly factor 1 (CAF1) also referred as nucleosome remodeling factor 55 (NURF55) (Fig. 4 and Table 1). E(z) contains a SET domain that catalyzes mono-, di-, and trimethylation of H3K27, which is a typical chromatin silencing mark. The ESC induces the enzymatic activity of E(z); however, NURF55 or CAF1 and Su(z)12 are important for nucleosome binding. PRC2 complex contains a distinct form of an additional protein polycomb-like (PCL). PCL protein is a classical PcG, which has been purified from Drosophila embryo which exists in association with E(z), Su(z)12, and ESC [93]. PCL protein is found at polycomb sites on polytene chromosome that is required for polycomb silencing of homeotic genes (Hox genes). Complete loss or mutation in PCL results in the activation of Hox genes and loss of H3K27me3 mark at target gene [106]. These five protein components are primarily responsible for trimethylation around PREs of H3K27, essential to sustain the repressed state (Fig. 5) [107]. In Drosophila, all main morphogenetic pathways are controlled by polycomb group genes, and these silencing are characterized by the formation of highly trimethylated of H3K27. The localization of PcG proteins to PREs has been investigated by genome wide high-resolution mapping [108].

Conversely, in mammals, the representation is more complex as PRC2, PRC3, and PRC4 complexes have been characterized biochemically, and their differences rely on the presence of different isoforms of embryonic ectoderm development (EED) (homolog to ESC) [109]. In mammals, the PcG proteins contain three primary core components, Su(z)12, EED, and enhancer of zeste homolog 2 (EZH2) or its homolog EZH1. EZH1 and EZH2 are the components of PRC2 complex that catalyzes mono-, di- and trimethylation of H3K27 [110, 111]. The trimethylation of H3K27 can act as a docking site for the protein subunit of PRC1, more specifically, the chromobox protein homolog (CBX) that provides a schematic mechanism for the recruitment of PRC1 to the target genes. The CBX forms the core of PRC1 together with one member of the human polyhomeotic (HPH) family (HPH-1 and HPH-3), ring fingers family (RING-1) (RING-Ia and RING-Ib), and polycomb group ring fingers family (PCGF) (PCGF-I to 6). Further, this complex catalyzes the mono-ubiquitination of histone-2A at lysine-119 (H2AK119ub) through RING-1 family E3 ligases, i.e., RING-1a and RING-1b [112, 113].

#### Pho-repressive complex

The only PcG proteins that bind directly to the DNA are Pleiohomeotic (PHO), and its closely related homolog PHO-like (PHOL). The PHO and PHOL are Drosophila homologs of the mammalian factor Yin-Yang 1 (YY1) and Yin-Yang 2 (YY2), respectively (Figs. 4 and 5 and Table 1) [103, 114]. The PRC1 and PRC2 have been functionally well characterized in Drosophila and mammals; however, PHO and PHOL have not been observed as an important component of PRC1 and PRC2 [91, 115–117]. Instead, PHO has been purified to exist in two different complexes in Drosophila. One of them is associated with chromatin remodeling machine, i.e., INO80. The second complex includes MBT-domain protein (SFMBT) involved in the silencing of homeotic genes. The repeats of MBT domain specifically bind to mono and dimethylated H3K9 and H4K20, respectively [96, 118, 119]. Different analysis of PHO and Pho-repressive complex (Pho-RC) at selected target genes have showed that it was specially localized at PRE sequence, and many of them are co-occupied by PRC1 and PRC2 complexes [89, 120, 121].

## HIV-1 latency and PcG proteins

The establishment of HIV-1 latency occurs by two ways. Firstly, the PcG proteins particularly PRC2 mediate mono-, di-, and trimethylation of H3K27. These repressive marks dominate the viral transcription in early phase of infection. In the second phase, the PRC2-mediated viral latency from an ongoing active HIV-1 infection [34]. In fact, PRC2 acts as a binding scenery for various DNA and histone-modifying enzymes including histone deacetylases [122], the SWItch/sucrose non-fermentable (SWI/SNF) bromodomain component containing Brd-7 [123] and DNA methyltransferase 1 (DNMT-1) [76]. The HIV-1 5′-LTR contains promoter and enhancer elements which induce HIV transcription by host transcription factors, while DNA methylation at 5′ LTR together with chromatin conformations restrict HIV reactivation characterizing a significant mechanism of latency maintenance [90, 124–126]. It has been observed that PRC1 and PRC2 are tightly related to control of HIV-1 latency, and the breaking agents of these complexes may be helpful to reactivate HIV from its latent reservoirs (Table 2) [127, 128].

**Table 2:** Cross talk between PcG proteins and HIV-1 in the maintenance of viral latency

Polycomb group protein	PcG complex	HIV-1 proteins	Biochemical interaction
Embryonic ectoderm development (EED)	PRC-2	MA, IN and Nef	Induces antiviral activities at the last stage of HIV-1 replication.
Phosphorylated enhancer of Zeste (p-EZH2)	PRC-2	Tat	Induces HIV-1 latency through Akt signaling pathway.
Retinoblastoma binding protein 4 (RbAp48/RBBP4)	PRC2	HIV-1 5′ LTR	Inhibits the production of viral particles at the transcriptional level.
Enhancer of Zeste 2 (EZH2)	PRC2	HIV-1 5′ LTR	Induces repressive mark on H3K27me3.
Suppressor of Zeste 12 (Su(z)12)	PRC2	HIV-1 5′ LTR	Catalyzes trimethylation in constitutive heterochromatin on H3K9 and H3K27.
Ying Yang 1 (YY1)	PhoRC	5′ LTR	Represses HIV-1 transcription and viral production.
BMI1 and RING1A	PRC1	5′ LTR	Regulate HIV-1 latency. Catalyze ubiquitination at H2K119
CBX	PRC1	5′ LTR	Binds to H3K27 trimethylation marks produced by PRC2 and induces E3-ligase activities.

### Enhancer of Zeste 2

Enhancer of Zeste homolog 2 (EZH2) is a member of PRC2 that contains histone methyltransferase activities, and it also acts as an epigenetic regulator with critical consequences of promoting HIV-1 gene silencing. The enzymatically active EZH2 is responsible for catalyzing methylation (mono, di, and tri) of H3K27, while the other three subunits such as retinoblastoma protein-associated protein 46/48 (RbAp 46/48), EED, and Su(z)12 promote chromatin compaction by enabling the enzymatic activities of EZH2 [129]. In HIV-1 latent reservoir, particularly in resting CD4+ T cell, the silencing of HIV-1 gene expression has been linked to the increased expression of EZH2. Their activities are upregulated by different mechanism such as signaling pathway, post-transcriptional modifications, highly reactive oxygen species, miRNA, and transcriptional factors; while on the other hand, they are downregulated by Akt signaling pathway which inhibits EZH2 activities and thus reactivate the HIV-1 from latency [130, 131]. Further, the presence of histone lysine methyltransferase component of PRC2 (EZH2) and Suv39h1 plays a unique role in the HIV-1 gene silencing. Remarkably, 40 and 5% reactivation of HIV-1 latent reservoirs are observed upon EZH2 and Suv39h1 knockdown, respectively [77, 79]. Recently, it has also been reported that PRC2 plays a critical role in the establishment of HIV-1 latency. Two latency models, i.e., HeLa/LTR-luciferase and U1 cells have been used to evaluate the function of PRC2 in the regulation of HIV-1 latency. Knockdown of PRC2 components: EZH2 and Su(z)12, reactivate HIV-1 from latent reservoirs [34]. Moreover, the repression of Su(z)12 increases sensitivity of HIV-1 Tat, and vice versa. Similar results were observed in U1 cells as viral transcription was enhanced in EZH2 and Su(z)12 knockdown cells [34]. Some other reports have also revealed that EZH2, the enzymatic component of PRC2, performs a key role in HIV-1 post-integration latency [132].

### Embryonic ectoderm development

The human embryonic ectoderm development (EED) is a major component of PRC2 and of the superfamily of WD-20 repeats also referred as WAIT-1 in human. Initially, EED was identified as cellular partner of HIV-1 matrix protein (MA), but later on, their interaction was also found with HIV-1-negative regulatory factor (Nef) and HIV-1 integrase (IN) [132]. The interaction of EED with IN favors the oligomerization of IN, which in turn enhances the HIV-1 integration process [133]. The interaction of EED with viral proteins inhibits HIV-1 maturation and its release thus indirectly promotes HIV-1 latency [134]. In human, four different forms of EED have been identified due to different translational initiations at particular codons for Val-1, Val-36, Met-95, and Met-110, related to the isoform EED-1, EED-2, EED-3, and EED-4, respectively [135, 136]. A moderate antiviral activity is associated with EED isoform EED-3 and EED-4 in the early phase of infection, while a strong negative effect on HIV-1 replication has been observed at late phase of viral infection [134]. EED-3 and EED-4 do not inhibit the expression of Gag protein, but instead it may interfere with viral packaging and genome assembly [134]. Moreover, EED together with B lymphoma Mo-MLV insertion region 1 (BMI-1) and RING-2 induces HIV-1 gene silencing by catalyzing trimethylation and ubiquitination at H3K27 and H2A, respectively [132]. Following the knockdown of polycomb genes such as bmi and eed reactivates HIV-1 from latent reservoirs. This reactivation is associated with decreased H3K27me3 and H2AK119ub, which reveals that these repressive epigenetic marks control HIV-1 latency [127].

### Suppressor of Zeste 12

Suppressor of Zeste 12 (Su(z)12) is an important component of EZH2 and EED complex, which is necessary for H3K27me3 and H3K9me3 in facultative and constitutive heterochromatin, respectively. However, they regulate H3K9me3 in an EZH2-independent manner [137]. Su(z)12 is also an important component of PRC2, which stabilizes EZH2 and hence enhances the HMTase activity of the complex. It contains two stretches of conserved amino acids that consist of C2H2 zinc finger-binding domain and C-terminal Vrn2-Emf2-Fis2-Su(z)12 (VEFS) domain required for the interaction between Su(z)12 and EZH2 [106]. Further, the region spanning residues of Su(z)12 (79-91) can act as the binding surface for RbAp46/48 [138]. However, mutation in Su(z)12 leads to strong homeotic transformation and lethality in Drosophila. Similarly, if Su(z)12 is missing in mice, it dies during embryogenesis at early post-implantation stage [139]. In mammalian cells, the knockdown of Su(z)12 causes demethylation of both H3K27me3- and H3K9me3-repressive marks and alters the distribution of HP1α [140]. Moreover, the genetic knockdown of Su(z)12, RbAp 46, and EED in Jurkat T cells is associated with proviral reactivation indicating that Su(z)12 and EED are also crucial for the methyltransferase activity of EZH2. Further, the downregulation of Su(z)12 leads to enhanced sensitivity of Tat-mediated transactivation of HIV-1 LTR. All of these components of PRC2 are recruited at HIV-1 promoter and impose H3K27me3 mark that silences the driving transcription of HIV-1 [78].

### Retinoblastoma-associated protein 46/48

Retinoblastoma associated protein 46 and 48 are also referred as RBBP7 and RBBP4, respectively. They are present in the CAF1 and play a critical role in the chromatin assembly [141]. Retinoblastoma-associated protein 46/48 (RbAp 46/48) is equivalent to 50 kDa WD repeat protein that is highly homologous to histone chaperones. It is present in HDAC and PcG complexes, therefore playing an important role in the establishment and maintenance of chromatin structure [142]. RbAp 46/48 recruits PRC2 complex to nucleosomes by binding to histone H3-H4 heterodimers, although it is not required for HMTase activity of EZH2, but both protein 46 and 48 are the components of PRC2 and remain unclear whether they have diverse function in the context of PRC2 [143]. Similarly, their role in the establishment of HIV-1 latency has not been well studied to date [144]. Recent studies conducted by Wu group have demonstrated that RbAp 46/48 inhibits HIV-1 production at the transcriptional level. The knockdown of RbAp 46/48 markedly enhance the production HIV-1 particles which clearly indicates that the RbAp 46/48 can act as transcriptional inhibitors of HIV-1 transcription. This may prompt the generation of specific therapies and may use as new drug targets in purging HIV-1 latent reservoirs [145].

### Ying Yang factor 1

YY1, a member of PcG proteins, is known as NF-E1, UCRBP, and CF1. It is a zinc finger-containing transcriptional regulator and ubiquitous cellular factor that plays a critical role in both activation and repression of gene, depending upon the promoter context. YYI was initially cloned and characterized by two independent groups at the same time [146, 147]. Over the last 25 years, YY1 was extensively characterized and became a rigorous focus of study due to its highly conserved sequence and ubiquitous in nature [148, 149]. Another cellular factor LSF (late SV40 factor) is known as CP-2, LBP-1c, or UBP-1 binds to 5′-LTR (−10 to +27) and recruits YY1 to LTR through its zinc finger domain. YY1 and LSF cooperate to form a complex called repressive complex sequences (RCS) which recruit HDAC1 that specifically and synergistically represses HIV-1 LTR expression and viral production by maintaining nuc-1 in hypoacetylated state [150, 151].

## Epi-inhibitors and HIV-1 latency: an area of pharmaceutical targeting

**Fig. 6:**
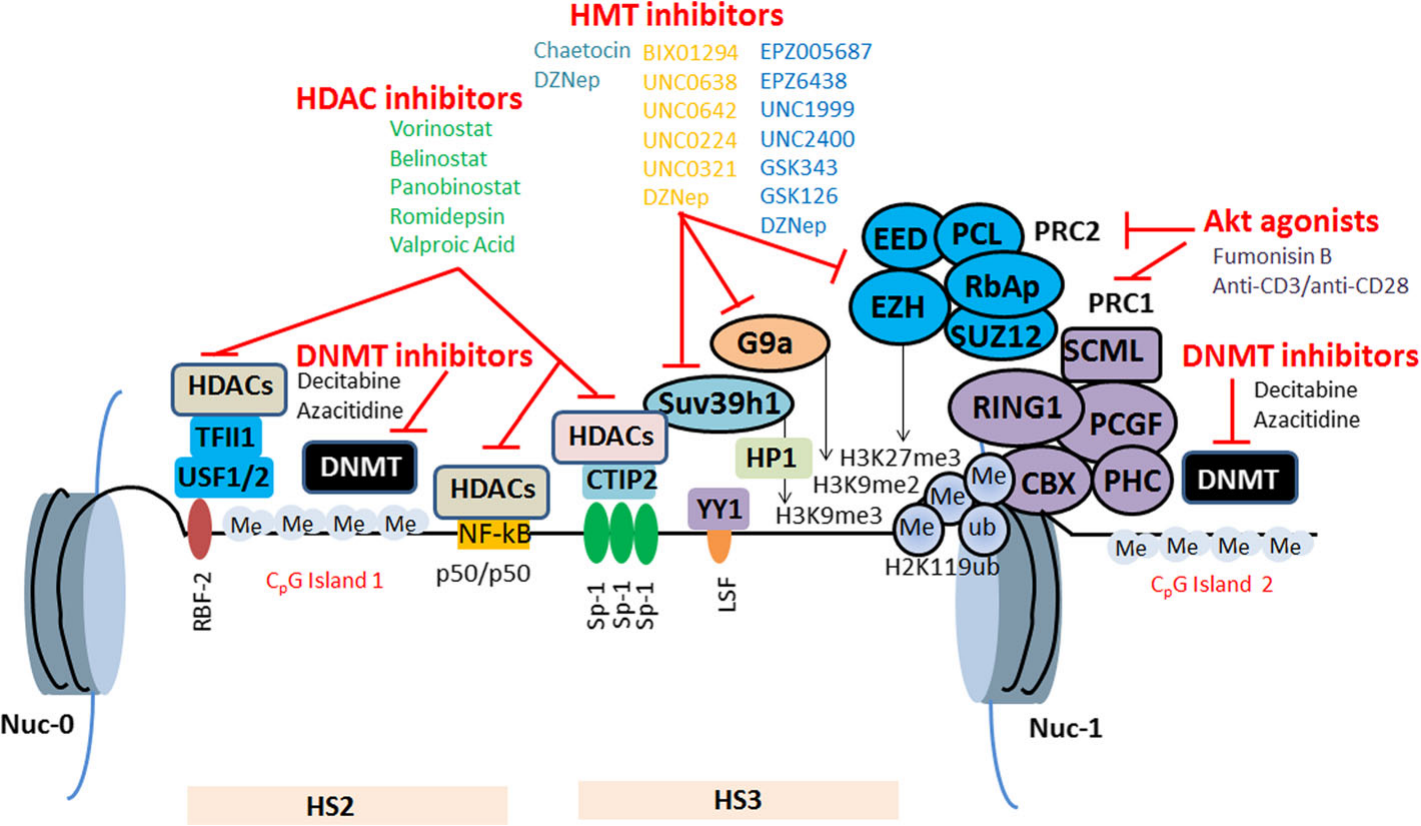
Molecular mechanism of HIV-1 proviral latency and strategies to disrupt latency by epi-inhibitors. The HIV-1 LTR is flanked by nuc-0 to nuc-1. During HIV-1 latency, nuc-1 is epigenetically silenced by several mechanisms. PRC2 recruits at nuc-1 and deposit the trimethylation marks onto H3K27. The repressive mark is recognized by CBX protein of PRC1. RING1, a component of PRC1, adds ubiquitination marks at H2K119. Nuc-1 is also epigenetically silenced by several transcription factors such as YY-1, CTIP-2, NF-kB p50/p50, homodimers. The corepressor CTIP-2 binds to Sp1 transcription factor at three sites in viral promoter and recruits HDACs and HMTs. Suv39h1 trimethylates H3K9 resulting in the recruitment of HP1. The HMT G9a mediates dimethylation of H3K9, which is also implicated in HIV-1 latency. The viral promoter is hypermethylated by DNMT at two CpG islands. PRC2 also recruits DNMTs promoting more silenced chromatin state. Various compounds have been proposed to reactivate the HIV-1 from latency including HMTIs (pyridone 6, chaetocin, UNCO638, UNC0642) to target HMTs (PcG of proteins, Suv39H1, G9a), HDACIs (panobinostat, romidepsin, valproic acid, vorinostat) to target hypoacetylated viral promoter, and DNMTIs (decitabine, 5-azacitidine) to target DNA methylation and Akt agonist to downregulate the PRC2

HIV-1 latency is controlled by a wide-range of factors and thus to wipe out HIV latent reservoirs may require multiple strategies. The shock-and-kill strategy has emerged to deal with this perplexing problem, i.e., activation of latent reservoirs from infected cells in combination with intensified cART; therefore, the eradication of HIV-1 latent reservoir pool may be accomplished [43, 152]. Furthermore, the complete elucidation of HIV-1 silencing in the cellular latent reservoirs is a considerably challenging task in HIV-1 eradication strategies. In this way, the recruitment of HMTs, HDACs, and DNMTs may influence the discovery of new drug targets and therapeutic breaks (Fig. 6).

Numerous latency reversal compounds have been explored which transcriptionally reactivate HIV-1 from its latent reservoir. These LRAs could be categorized into four different epigenetic drugs: histone methyl transferase inhibitors (quinazoline derivatives); DNA methyl transferase inhibitors (decitabine, azacitidine, CP-4200, S-100); histone deacetylase inhibitors (vorinostat, romidepsin, panobinostat, valproic acid); and histone demethylase inhibitors (polyamine analogues) [23, 153, 154]. Until now, three different types of DNMT inhibitors, i.e., decitabine (dacogen), 5-azacitidine (vidaza), and panobinostat (farydak) and HDAC inhibitors, i.e., vorinostat (zolinza), belinostat (beleodaq), and romidepsin (istodax), have been approved for cancer therapy. Furthermore, a diverse portfolio of LRAs is under study and is currently at different pre-clinical and clinical stages as novel therapies for various conditions [155, 156]. Histone methyltransferases are also known as protein methyltransferases (PMTs) because they methylate non-histone protein as well. The histone methyltransferases are divided into three main categories, i.e., writers, readers, and erasers. Modifications are created by the writer enzymes, while reader proteins recognize these modifications and eraser enzymes remove the same modifications. HMTs are also involved in the repression of latent HIV-1, as discussed above, but the treatment of latently infected cells with HMTIs which target G9a, EZH2, Suv39h1, and H3K27me3 marks can lead to reactivation of silenced provirus at transcriptionally inactive viral promoter [78, 157]. In contrast to HDACIs and DMTIs, the search for HMTIs is still in its infancy. During last 4 years, different types of high-quality small molecule inhibitors have been discovered, i.e., UNC0638 and UNCO642 for G9a; EPZ-6438, GSK126, and EI1 for EZH2; UNC1999 for EZH2/EZH1; EPZ004777, SGC0946, and EPZ-5676 for DOT1L, and (R)-PFI-2 for SETD7. These molecules have been utilized in a number of animal-based disease models and cell-based studies [158–163].

BIX01294, a diazepin-quinazolinamine derivative, was reported firstly as a selective small molecule inhibitor of G9a- and G9a-like protein histone methyl transferase which was a great advancement in the field of histone methyltransferase inhibitors [164]. BIX01294 is active at the promoter of G9a target genes and can selectively reduce H3K9me2 and also reactivates HIV-1 latent reservoir in vitro [165]. Although, in cellular assays, it is toxic at concentration above 4.1 μM. Moreover, the optimization of this quinazoline (BIXO1294) scaffold has led to the development of other several cellular and chemical probes such as UNC0224, UNC0321, UNC0638, and UNC0642. However, probe UNCO638 and UNC0642 have been extensively characterized in a series of cellular, chemical, and biophysical assays. These inhibitors have not only exhibited high in vitro effectiveness and better selectivity but also displayed the targeted activities in cellular models. These chemical probes are a useful tool to interrogating the role of G9a in health and diseases in cell-based studies [164, 166–168]. These findings have opened the door of pharmacological inhibitions of histone methylation at H3K27, H4K20, and H3K9. In 2012, Bouchat et al. reported that HMTIs BIX01294, chaetocin, and the broad spectrum DZNep reactivate the latent reservoirs in the different latently infected cell lines [165]. In HAART-treated patients with unnoticeable viral load, chaetocin (Suv39h1 inhibitor) reactivates the HIV-1 from latency in CD4+ T cells isolated from HIV-1-infected patients, while BIX01294 induces 80% recovery in resting memory CD4+ T cells isolated from an HIV-1-infected patient. However, chaetocin and BIX01294 cannot be safely administered to human [77, 165]. Another study has shown that chaetocin causes 25-fold induction of latent HIV-1 reservoir, without causing toxicity and T cell proliferation or expansion. The induction is associated with loss of methylation and accumulation of acetylation marks of H3K9 at HIV-1 promoter indicating that a significant chromatin remodeling is regulated by chaetocin [77, 169].

Pyridone 6 or compound 6, a small molecule and inhibitor for EZH2, is another major expansion in the field of HMT inhibitors [170]. The synthesis of this probe or small molecule inhibitor has led to the discovery of many other chemical probes such as EPZ-6438, GSK126, GSK343, EI1, UNC1999, and UNC2400 [171, 172]. The mentioned inhibitors of EZH2 have displayed robust activities in target cells and selectively reduced the trimethylation mark of H3K27 in a number of wild and mutant cell lines of EZH2 [172]. Prominently, EPZ-6438 and GSK126 have shown high efficacy rates in vivo. The intraperitoneal and oral administration of GSK126 and EPZ-6438 has led to drastic reduction in tumor volume and significant improvement in the survival of more aggressive KARPAS-422 tumor in xenografts mouse model [173]. In 2013, the first EZH2 inhibitor entered into human clinical trials was EPZ-6438 while GSK126 has advanced to phase 1 clinical trials for the treatment of various diseases [172, 174–176]. Friedman et al. reported the efficacy rates of broad spectrum HMTIs DZNep that mainly target EZH2 component of PRC2 and can purge HIV-1 from the latent reservoirs [76]. In comparison to azacitidine, decitabine, chaetocin, and BIX01294, DZNep is highly potent in reactivating the viral reservoirs from latency. Unfortunately, DZNep is cytotoxic at concentration required to purge latent HIV-1 reservoir [77]. Recently, two novel inhibitors of EZH2, i.e., DEC_42 and DEC_254, have been discovered by using a combined in silico screening and experimental study. These two compounds are different from other EZH2 inhibitors by exhibiting new molecular structure. Moreover, their activities are very low (IC_50_ values of 22.6 and 10.3 μmole/L, respectively) and require further optimization [177]. These findings clearly indicate that PcG proteins are associated with maintenance of HIV-1 latency (Table 2). It may also give an important insight in developing novel anti-HIV drugs that disrupt PcG proteins mediating gene silencing in HIV-1 reservoirs. However, it is important to mention that epi-drugs have some side effects and should all be evaluated carefully for the treatment of HIV-1 infections and various other diseases [155, 165]. FDA has approved various epi-drugs, and there is still potential for improvement as these drugs are relatively unstable and can have some side effects, e.g., at high dose, 5-azacytidine causes neutropenia [178–180]. While, the administration of vorinostat (SAHA) is associated with anorexia, anemia, hyperglycemia, thrombocytopenia, fatigue, nausea, and ECG abnormalities [178, 180]. Similar toxic side effects have also been observed with romidepsin and belinostat [181]. However, the long-term safety of epi-drugs has not been assessed in HIV-1-positive people.

## PcG-mediated epigenetic silencing and novel HIV-1 reactivation strategies under cART

The concept of eliminating or reactivating HIV-1 from latency was initially demonstrated by T cell activators such as IL-2 and anti-CD3 antibody, but this approach was associated with unacceptable toxicity of T cell proliferation and expansion [182, 183]. In this regard, epi-inhibitors are capable of reversing HIV-1 latency without causing significant toxicities [165, 184]. The shock-and-kill strategy is characterized by the use of epi-inhibitors to reverse HIV-1 latency and reactivate the HIV-1 from target cells such as CD4+ T cells and monocytes/macrophages that are supposed to be cleared by viral cytopathic effect [156, 185, 186].

**Fig. 7:**
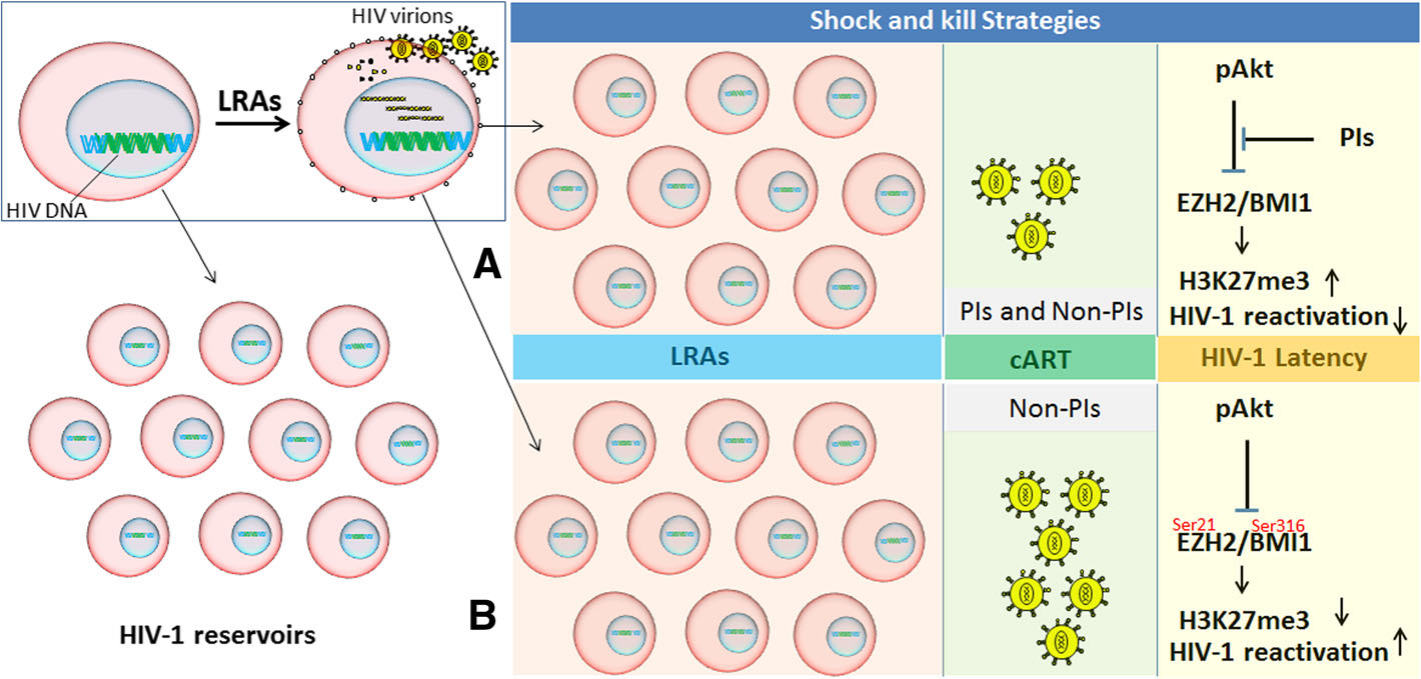
Novel clinical parameter in shock-and-kill strategy to target HIV-1 latency. The figure shows the total pool of cells latently infected with HIV (left side) and how LRAs reactivates HIV-1 from latency (enlargement). Panels **a** and **b** are schematic diagrams of novel shock-and-kill strategies with various degree impacts of PIs and non-PIs on the reactivation of viral reservoirs. **a** Illustrates the reactivation of HIV-1 by LRAs in the presence of cART regimen. The presence of PIs in the standard cART regimen inhibits the Akt signaling which in turn enhances EZH2-mediated H3K27me3 and HIV-1 latency. **b** Illustrates the reactivation of HIV-1 LTRs in the presence of cART-containing non-PIs. Akt phosphorylates EZH2 and BMI1 at Ser 21 and Ser 316, respectively. It impairs their functions which results in decreased H3K27me3 and H2A ub and thus strongly reactivates HIV-1 from its latent reservoirs

Why current LRAs are not decreasing the pool of latent HIV-1 reservoirs? There are many factors that need to be considered. Firstly, epi-inhibitors do not activate all the HIV-1 latent reservoirs while strong cytotoxic T lymphocytes (CTLs) response are required to clear the reactivated pool of HIV-1 reservoirs, but impaired CTLs response has been observed [187–189]. Secondly, the data from various clinical trials have shown that LRAs insufficiently reactivate HIV-1 latent reservoirs [187, 190]. At present, we do not have the answer—why LRAs partially reactivate HIV-1 from latency? Further, it is unclear whether the shock induced by LRAs is influenced by cART or not. Besides interfering with polyprotein processing of immature virions, protease inhibitors (PIs) can influence several aspects of HIV-1 disease progression. In this regard, Kumar et al. recently reported that PIs regulate HIV-1 latency through Akt signaling [191]. They found that PIs but not non-PIs can limit LRA-mediating reactivation of HIV-1 latent reservoirs. The same group demonstrated that PIs block the reactivation of HIV-1 in resting CD4+ T cells isolated from chronically infected aviremic patients [192]. In addition to HIV-1 disease progression, Akt signaling also regulates the chromatin remodeling through PcG proteins [193]. In resting CD4+ T cells, the EZH2, a component of PRC2 is associated with H3K27me3 and thus enhances HIV-1 latency and so the activity of EZH2 can be regulated by Akt. Activation of Akt signaling phosphorylates EZH2 and inhibits its enzymatic activity, thus releasing the epigenetic silencing of HIV-1 promoter [193, 194]. In contrast to non-PIs, the administration of PIs during shock-and-kill strategies may enhance HIV-1 latency by blocking Akt signaling, and so, it increases the enzymatic activity of EZH2 (Fig. 7). In addition to EZH2, Akt also impairs the function of BMI1 by phosphorylating it at Ser 316. This Akt-mediated phosphorylation of BMI1 is associated with decreased H2A ubiquitination [195]. However, the impact of cART on Akt signaling and the reactivation of HIV-1 from latent reservoirs require further investigation and validation.

## PcG-mediated epigenetic silencing will set the stage for block-and-lock strategy?

Efforts for a sterilizing cure of HIV-1 infection have been focused on the “shock and kill” strategy [185]. However, this method has faced various challenges [187]. Recently, the block-and-lock strategy for functional cure of HIV-1 has been proposed [181]. This novel strategy aims to reinforce a deep state of latency by using latency-promoting agents (LPAs). Interestingly, a few recent findings indicate that this strategy is quite feasible [196–199].

**Fig. 8:**
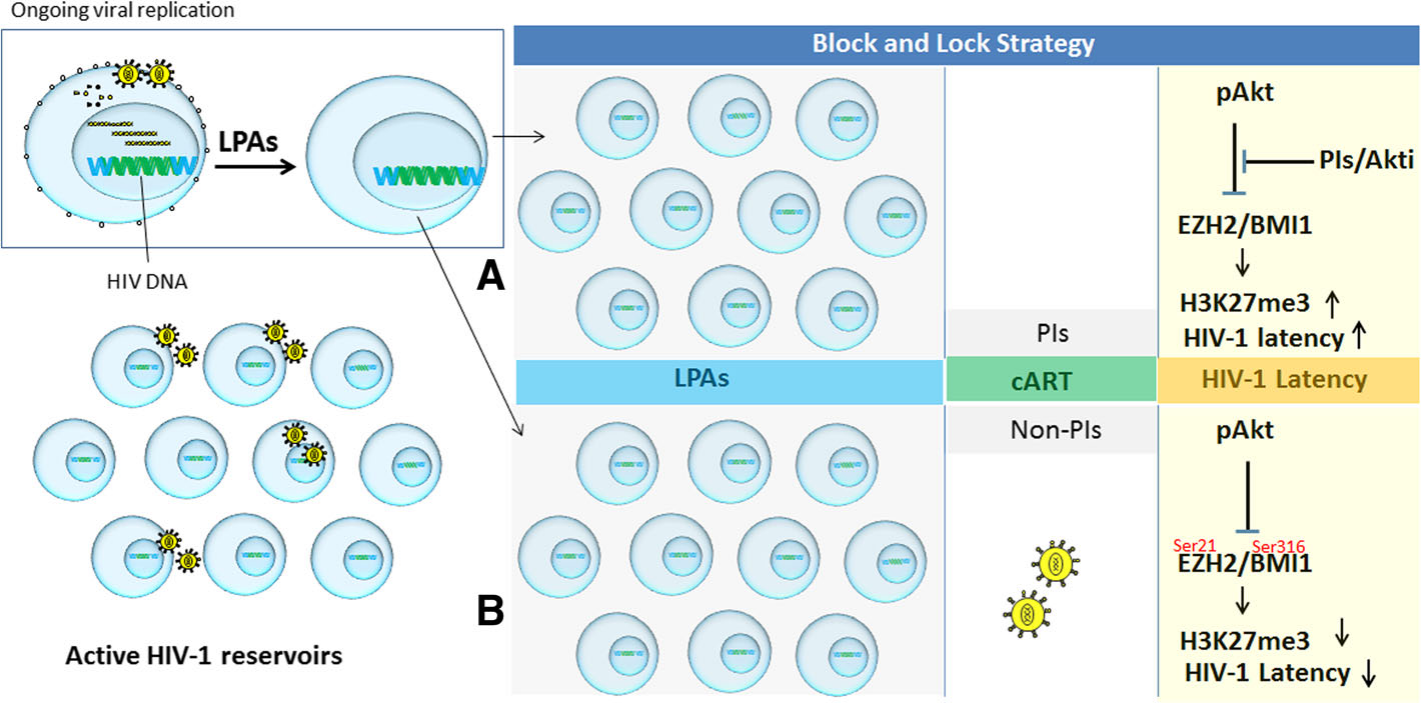
Novel clinical parameter in block-and-lock strategy to induce HIV-1 latency. The figure shows the ongoing viral replication from active HIV-1 reservoirs (left side) and how LPAs promote HIV-1 latency and suppress viral reactivation (enlargement). Panels **a** and **b** are schematic diagram of novel block-and-lock strategy with various degree impacts of PIs and non-PIs on the induction of viral latency. **a** Illustrates the suppression of HIV-1 replication by LPAs together with PIs. The presence of PIs and Akt inhibitors in the standard cART regimen inhibit the Akt signaling which in turn synergistically enhance EZH2-mediated H3K27me3 and HIV-1 latency. **b** Illustrates HIV-1 latency in the presence of cART containing non-PIs. Akt phosphorylates EZH2 and BMI1 at Ser 21 and Ser 316, respectively. It impairs their functions which results in decreased H3K27me3 and H2A ub and weakly suppresses HIV-1 from its latent reservoirs

In block-and-lock strategy, the role of PcG-mediated epigenetic silencing has become important. EZH2 is a core component of PRC2, and its SET domain catalyzes trimethylation of H3K27, a histone modification associated with transcriptional silencing [200]. Trimethylation of H3K27 recruits PRC1 to the chromatin which further reinforces the PcG-mediated epigenetic silencing [116]. Since, TrxG and PcG antagonize each other [201]. In this way, targeting the TrxG proteins may stimulate the PcG-mediated epigenetic silencing. Mixed lineage leukemia (MLL) is a mammalian homolog of the trithorax (Trx) protein found in *Drosophila melanogaster* [202]. Small molecules such as menin-MLL inhibitor (MI-2), pinometostat (EPZ-5676), and Flavopiridol (alvocidib), have shown promising efficacies in targeting MLL/Trx and represent potential therapeutic strategies [203]. Administration of these drugs will inhibit Trx-mediated H3K4me2 and stimulate the PcG-mediated epigenetic silencing that may enhance the HIV-1 latency. In addition, the activity of PcG can be modulated through Akt signaling [194, 195]. Activation of Akt signaling inhibits PcG-mediated trimethylation of H3K27. So, it limits the HIV-1 silencing. Arguably, Akt inhibitors may provide a better and superior choice of drug in inducing the viral latency. Akt inhibitors may inhibit the Akt-mediated phosphorylation of EZH2 and may induce its enzymatic activity. Hence, enhancing the epigenetic silencing of integrated HIV-1 genome [193]. Moreover, Akt inhibitors may impair the Akt-mediated phosphorylation of BMI-1 [195]. Akt inhibitor may induce H2A ubiquitination and may promote epigenetic silencing of HIV-1 promoter. In addition, cART may impact the block-and-lock strategy of HIV-1 cure, since PIs inhibit Akt signaling and suppress HIV-1 reactivation from latency [191, 192]. The use of PIs or Akt inhibitors together with LPAs may synergistically induce viral latency and may contribute to functional cure of HIV by preventing viral reactivation from latent reservoirs (Fig. 8).

## Conclusion

Intensive work has been done by the scientific community to investigate the molecular mechanisms involved in the establishment of HIV-1 latency. Improved understanding in viral persistence has paved the way for novel strategies to limit the HIV-1 reservoirs. One approach for the eradication of HIV-1 reservoirs is the application of anti-latency agents or latency-reversing agents (LRAs) to force the reactivation of HIV from latency at various levels. In the recent past, combination of drugs that alter chromatin status have already been revealed to generate a synergistic reactivation of HIV-1 from its latent reservoirs. Soon, it became clear that the induction of latent viral reservoirs by the shock-and-kill strategy may not be sufficient to clear latently infected cells, but the recognition of viral antigens by the immune cells specifically broad CTLs response may be required to identify and clear the latently infected reservoirs. Histone methylation, acetylation, and DNA methylation have been under investigation for drug design, and many of its inhibitors are FDA-approved for numerous disorders such as cancer. More recently, compounds targeting EZH2 and LSD are under investigation to modulate the epigenetic markers regulating HIV-1 latency. Due to the heterogeneous nature of cellular reservoirs, shock-and-kill strategy still requires a hard and long road to achieve a sterilizing or functional cure of HIV. Anyhow, other therapeutics strategies and targets should be explored. Suppression of ongoing viral replication from active viral reservoirs is one strategy that has recently gained considerable attention. In this regard, PcG proteins may provide a promising novel targets for the induction of HIV latency. The establishment of deep latency through PcG could prevent viral rebound when cART is interrupted. A combination of Akt inhibitors together with PcG proteins may represent an interesting approach for future therapeutic intervention and a functional cure of HIV-1.

## Abbreviation

AIDS: Acquired immunodeficiency syndrome
APOBEC3G: Apolipoprotein B mRNA editing enzyme catalytic subunit 3G
BMI1: B lymphoma Mo-MLV insertion region 1
CAF1: Chromatin assembly factor 1
cART: Combination antiretroviral therapy
CBX: Chromobox protein homolog
CDK9: Cyclin-dependent kinase 9
CNS: Central nervous system
CTIP2: COUP-TF-interacting protein 2
CTLs: Cytotoxic T lymphocytes
DNMT: DNA methyltransferase
DNMTi: DNA methyltransferase inhibitor
dRAF: dRING-associated factors
DZNep: 3-Deazaneplanocin A
EED: Embryonic ectoderm development
ESC: Extra sex combs
EZ: Enhancer of zeste
EZH2: Enhancer of zeste homolog 2
H3K27: Histone H3 on Lys27
HDAC: Histone deacetylase
HDACi: HDAC inhibitor
HIV-1: Human immunodeficiency virus type 1
HMT: Histone lysine methyltransferase
HMTI: Histone methyltransferase inhibitor
HP1: Heterochromatin protein 1
HPH: Human polyhomeotic
LRA: Latency-reversing agent
LSD1: Lysine-specific demethylase 1
LSF: Late SV40 factor
LTR: Long terminal repeat
NF-kB: Nuclear factor-κB
NURF55: Nucleosome remodeling factor 55
PC: Polycomb
PCAF: P300/CBP-associated factor
PcG: Polycomb group
PCL: Polycomb-like
PH: Polyhomeotic
PHO: Pleiohomeotic
PHOL: PHO like
Pho-RC: Pleiohomeotic repressive complex
PI: Protease inhibitor
PRC1: Polycomb-repressive complex 1
PRC2: Polycomb-repressive complex 2
PREs: Polycomb response elements
PSC: Posterior sex combs
PTEFb: Positive transcription elongation factor b
RbAp: Retinoblastoma-binding protein
RIZ: Retinoblastoma protein-interacting zinc-finger
SCE: Sex comb extra
SCM: Sex comb on midleg
SFMBT: Scm-like with four MbT domain-containing protein 1
SMYD: SET and MYND domain
SP1: Specificity protein 1
Su(z)12: Suppressor of Zeste 12
Suv39h1: Suppressor of variegation 3–9 homolog 1
SWI/SNF: SWItch/sucrose non-fermentable
TAR: Trans-activation response
Tat: Trans-activator of transcription
TRIM5α: Tripartite motif-containing protein 5α
TrxG: Trithorax group proteins
WHO: World health organization
YY1: Ying-yang 1
